# Super-tension-reduction suture versus conventional closure for secondary closure of infected abdominal incisions

**DOI:** 10.3389/fsurg.2026.1829502

**Published:** 2026-05-20

**Authors:** Changkai Zhou, Lu Wang, Zhenhua Gong, Tie Xiang

**Affiliations:** Department of Burn and Plastic Surgery, Nantong First People’s Hospital, Nantong, China

**Keywords:** abdominal wound repair, complication, super-tension-reduction suture, surgical site infection, wound healing

## Abstract

**Background:**

Surgical site infection is a common complication after abdominal surgery and is associated with delayed wound healing, prolonged hospitalization, and increased healthcare costs. Secondary closure of infected abdominal incisions after debridement and negative pressure wound therapy remains challenging because tissue edema, inflammation, and skin retraction often lead to excessive wound tension. Super-tension-reduction suture has been proposed to redistribute wound tension and improve tissue perfusion, but its effectiveness in abdominal wound salvage remains unclear.

**Methods:**

We performed a retrospective cohort study of patients undergoing abdominal wound salvage for superficial/incisional surgical site infection between January 2020 and May 2024. Patients were categorized according to the final closure technique: standard interrupted suture or super-tension-reduction suture using a modified buried vertical mattress technique. Patient demographics, operative characteristics, and postoperative outcomes were collected. Primary outcomes included recurrent surgical site infection, wound breakdown, hematoma, seroma, fat necrosis, and reoperation. Secondary outcomes included operative duration, time to drain removal, time to suture removal, and length of hospitalization. Propensity score matching was used to mitigate the selection bias. Multivariable binary logistic regression identified significant predictors of complications.

**Results:**

We included 89 patients (standard interrupted suture group: 39; super-tension-reduction suture group: 50). In the unmatched cohort, super-tension-reduction suture was associated with lower rates of recurrent surgical site infection (6.0% vs. 23.1%, *P* = 0.028) and reoperation (4.0% vs. 17.9%, *P* = 0.039), alongside shorter hospitalization (17.5 vs. 21 days, *P* < 0.001), despite a modestly longer operative time (24 vs. 20 min, *P* < 0.001). After 1:1 propensity score matching (*n* = 58), super-tension-reduction suture group maintained a significantly lower overall complication rate (17.2% vs. 41.4%, *P* = 0.043). Crucially, multivariable logistic regression identified the super-tension-reduction suture as an independent protective factor against overall complications (aOR: 0.22, 95% CI: 0.08–0.59, *P* = 0.003) and recurrent surgical site infection (aOR: 0.21, 95% CI: 0.05–0.85, *P* = 0.028).

**Conclusion:**

The super-tension-reduction suture was associated with favorable clinical outcomes in abdominal wound salvage. Compared with conventional closure, it was associated with lower overall complication rates despite a modest increase in operative time. While clinically promising, these observational associations warrant further validation in prospective, randomized trials.

## 1 Introduction

Surgical site infection (SSI) remains one of the most debilitating postoperative complications in abdominal surgery, significantly increasing morbidity and healthcare expenditure (1). Abdominal surgeries, often involving hollow viscera and potential contamination risks, are associated with a high incidence of incision infections (15%–25%), presenting a challenging, complex, and potentially life-threatening issue for surgeons (2, 3).While debridement and negative-pressure wound therapy (NPWT) have streamlined wound bed preparation (4), the subsequent secondary closure of these defects presents a profound biomechanical challenge. Persistent tissue edema and severe skin retraction inevitably result in extreme mechanical tension upon direct fascial and epidermal approximation. Crucially, excessive wound tension acts as a pathological mechanical stimulus that obliterates local microcirculation. This induced ischemia and hypoxia not only disrupt collagen metabolism and inhibit fibroblast proliferation but also severely compromise the local innate immune response, creating a susceptible microenvironment for recurrent bacterial colonization (5).

Therefore, mitigating incisional tension is not merely a cosmetic requirement but a fundamental biological necessity for infected wound salvage. Recently, the concept of super-tension-reduction suture (STRS)—an advanced tissue-approximation strategy designed to structurally redistribute excessive biomechanical loads from fragile wound edges into robust deep connective tissues—has emerged as a transformative approach (6). In this study, we operationalized the STRS concept utilizing a specific technique: the modified buried vertical mattress suture (MBVMS) (7, 8). Crucially, the unique three-dimensional architecture of this modified vertical technique not only minimizes epidermal strain but also effectively obliterates subcutaneous dead space—a well-known sanctuary for fluid accumulation and bacterial proliferation. Concurrently, it promotes the natural eversion of skin edges, maximizing dermal contact area to facilitate rapid microvascular reconnection. By preserving marginal skin perfusion and limiting the inflammatory response induced by mechanical stress (5), this specific STRS technique theoretically establishes an optimal microenvironment for wound healing. However, robust clinical evidence evaluating its translation from aesthetic practice to contaminated abdominal wound salvage remains limited. We hypothesized that modifying the biomechanical environment via the STRS(MBVMS) technique could disrupt the tension-ischemia-infection cycle, thereby being associated with reduced SSI recurrence and improved clinical outcomes compared to conventional layered closure.

## 2 Materials and methods

### 2.1 Patient selection

This study was approved by the Institutional Review Board (approval number: 2025-KT359-01) and conducted in accordance with the Declaration of Helsinki. We systematically reviewed the electronic medical records of all patients who underwent secondary surgical closure for abdominal surgical site infections (SSI) at our institution between January 1, 2020, and May 31, 2024. To ensure a homogeneous cohort, the patients were enrolled based on strictly defined criteria.

Inclusion criteria were:

- Aged 18–85 years.
- Postoperative superficial incisional SSI following abdominal surgery.
- Failure of conservative treatment requiring surgical reintervention.
- Secondary suture closure performed after debridement and NPWT.
- A minimum postoperative follow-up of 30 days or until complete wound healing adequately captured the short-term postoperative complications.

Exclusion criteria comprised:

- Abdominal wall infection complicated by systemic dissemination or unstable vital signs.
- Infection involving the organ/space or necrosis of the deep muscle layer.
- Comorbid necrotizing fasciitis.
- Infection of implants.
- Massive abdominal skin defects precluding direct secondary closure.

In this study, SSI classification was strictly defined according to the Centers for Disease Control and Prevention (CDC) guidelines (9). Our cohort exclusively included patients with “superficial incisional SSIs” that involved only the skin and subcutaneous tissue of the incision. Patients presenting with either “deep incisional SSIs” (involving deeper soft tissues, such as the fascial and muscle layers) or “organ/space SSIs” (involving any part of the intra-abdominal anatomy) were strictly excluded. This distinction is critical because deep fascial involvement and organ/space infections require fundamentally different source-control strategies, such as complex fascial debridement or intra-abdominal drainage, and are not amenable to superficial body wall reconstruction techniques such as STRS.

Eligible patients were categorized into two cohorts based on the final closure technique: the standard interrupted suture (SIS) group and STRS groups. The allocation to either group was not randomized but was determined by the attending surgeon's intraoperative assessment and evolving institutional practices. Conventional layered interrupted suturing was the standard approach used during the early phases of the study period. However, because the STRS technique has demonstrated significant advantages in biomechanical offloading, it has progressively become the preferred institutional protocol for wound management. Specifically, STRS is strongly indicated for patients exhibiting severe bilateral skin retraction, excessive wound tension, or where the conventional approximation induces visible ischemia at the skin edges.

### 2.2 Surgical technique

The precise timing of secondary surgical closure is strictly governed by a standardized institutional wound bed preparation protocol rather than a fixed chronological timeframe. Upon SSI diagnosis, all participants underwent immediate and aggressive surgical debridement of the infected incision followed by application of a closed negative-pressure wound therapy (NPWT) system. Targeted, culture-directed antimicrobial therapy has been initiated and continuously optimized based on serial bacterial cultures and sensitivity profiling.

The indication for secondary closure was continuously reevaluated during regular NPWT dressing changes. Patients were considered eligible for definitive closure only when they unequivocally met a strict triad of clinical and microbiological criteria: **(1) Macroscopic/Local Criteria:** Complete resolution of local acute inflammatory signs (erythema, induration), with the wound bed exhibiting 100% robust, highly vascularized granulation tissue, entirely devoid of macroscopic necrosis, slough, or purulent exudate. **(2) Microbiological Criteria:** Confirmed eradication or stable control of the primary pathogen objectively evidenced by negative consecutive wound swabs or tissue cultures prior to closure. **(3) Systemic Criteria:** Hemodynamic stability coupled with the normalization or significant downtrend of systemic inflammatory markers (e.g., white blood cell count and C-reactive protein), ensuring that the patient can tolerate the physiological stress of a high-tension fascial/epidermal approximation.

Once these biological conditions were met, the feasibility of primary wound closure was assessed in a manner similar to that used in the free-flap design. Specifically, following proper subcutaneous dissection over the deep fascia and wedge-shaped excision (10) to remove the fibrotic wound edges, the wound margins were approximated manually or with trial sutures to ensure closure without excessive tension. Final closure (SIS or STRS) was performed only after physical assessment.

The SIS group used a traditional layered interrupted suture technique. The subcutaneous superficial fascia was sutured using absorbable sutures to eliminate partial dead space, followed by interrupted suturing of the skin using non-absorbable or absorbable sutures.

**Figure 1:**
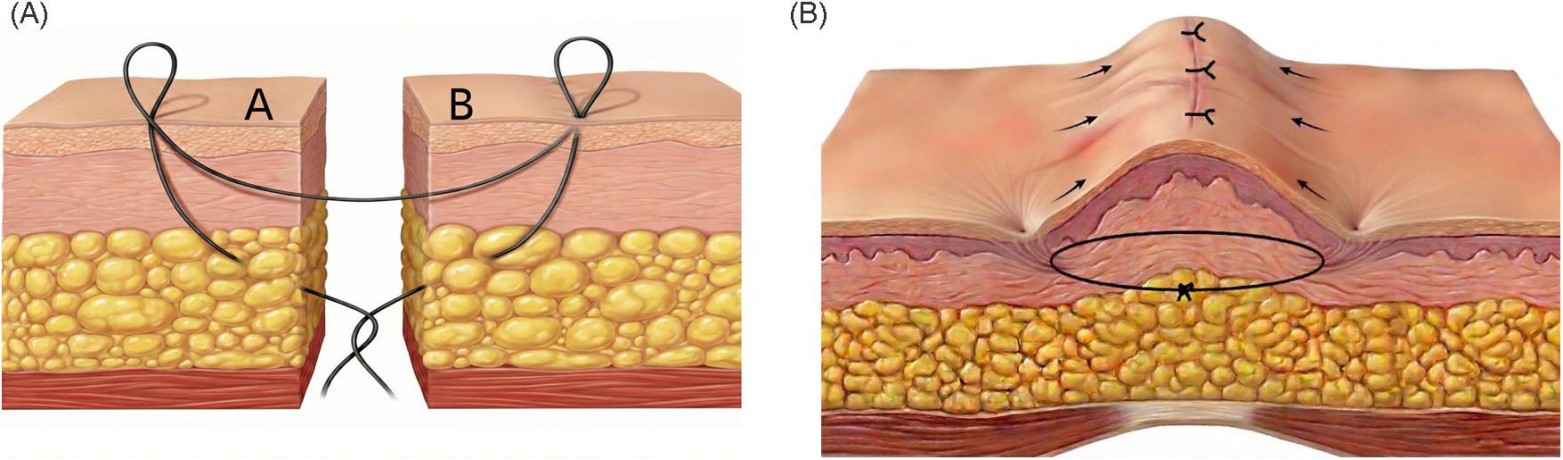
Schematic drawings of the subcutaneous super tension reduction suture before **(A)** and after suturing **(B)**.

**Figure 2:**
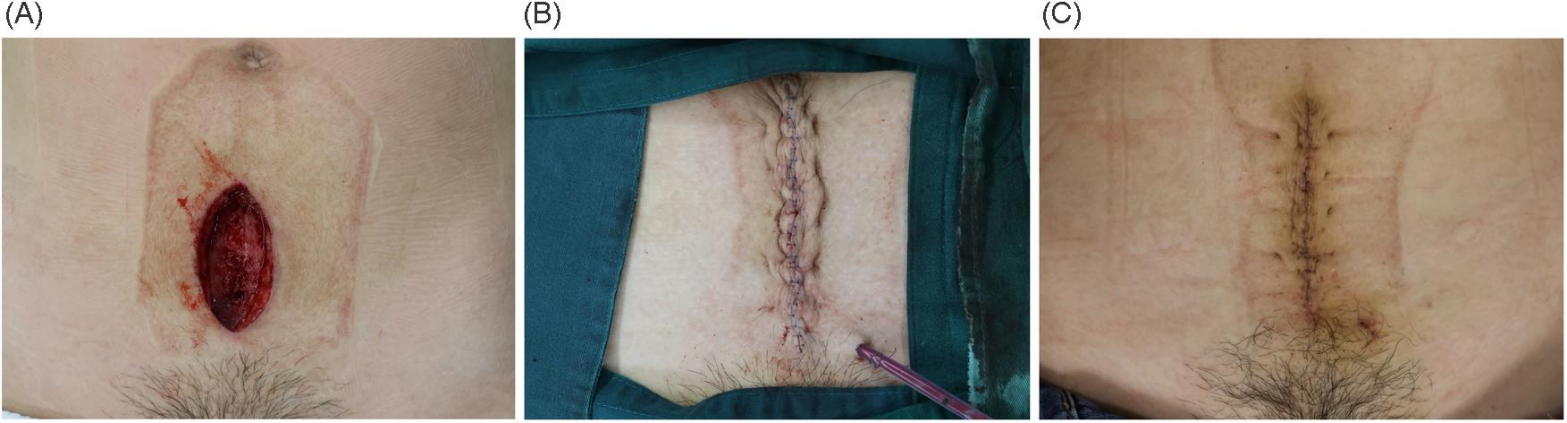
Images of a 35-year-old woman with abdominal SSI before **(A)** and after super tension reduction suturing **(B)**. The wound healed eventually **(C)**.

In the STRS cohort, wound closure was performed utilizing the percutaneous buried vertical mattress suture technique to achieve optimal biomechanical offloading. The procedure was meticulously executed through a precise sequence. First, the needle was inserted into one side of the wound (Side A), beginning in the deep reticular dermis. The needle was then passed laterally and brought out through the skin surface at an appropriate distance from the wound margin. Crucially, the needle was then reinserted into the exact same cutaneous puncture site, angled to pass through the superficial dermis, and brought out into the center of the wound. This trajectory was then repeated in a mirrored, symmetric fashion on the contralateral side (Side B). The needle entered the superficial dermis, exited the skin laterally, and was similarly reinserted into the same puncture site before angling downward to exit from the deep reticular dermis into the wound center (8, 11) (Figures 1A,B). Finally, the buried suture was securely tied in the deep tissue. Following the securement of these primary tension-relieving percutaneous sutures, standard interrupted cutaneous sutures were placed along the incision line to complete the final cutaneous repair. Crucially, because the underlying percutaneous mattress had already offloaded the biomechanical stress, these superficial sutures were tied with minimal tension. Their exclusive function was to achieve precise epidermal approximation and ensure a watertight biological seal, rather than to bear mechanical load (Figures 2A,B).

Subcutaneous drains were placed in all incisions postoperatively and removed if the drainage volume was <10 mL/day. Surgical incisions were covered with standard surgical dressings, which were changed regularly. Non-absorbable skin sutures were removed approximately 10–14 days postoperatively upon the surgeon's assessment of healing (Figure 2C).

### 2.3 Variables and outcomes

Patient data were extracted from the electronic medical record system, including demographics, smoking status, body mass index (BMI), comorbidities, ASA score, history of initial abdominal surgery, dimensions of the infected abdominal wound, bacteriological results, time interval from SSI onset to secondary closure, wound dimension and preoperative albumin levels.

The primary outcomes were postoperative complications included recurrent SSI, reoperation rate, wound dehiscence, hematoma, seroma, skin necrosis, and fat liquefaction. To minimize diagnostic ambiguity, the assessment of recurrent SSI was strictly standardized and evaluated in accordance with the Centers for Disease Control and Prevention (CDC) criteria (9) for site infections. Given that all patients had a prior history of infection, a “recurrent SSI” was explicitly diagnosed if any of the following clinical or microbiological events occurred within 30 days following the secondary closure: (1) **Clinical signs of severe inflammation:** New-onset or spreading erythema, localized induration, or increased pain at the incision site that clinically necessitated the initiation or escalation of targeted antibiotic therapy; (2) **Purulent discharge or collection:** Spontaneous purulent drainage from the closed incision, or the clinical necessity for bedside or operative abscess drainage/re-opening of the wound; (3) **Microbiological confirmation:** A positive bacterial culture obtained aseptically from wound fluid or deep tissue that demonstrated the same pathogenic profile as the initial infection. These assessments were conducted systematically by the attending surgical team. Evaluations were performed during daily inpatient rounds, at the time of suture removal (typically 10–14 days postoperatively), and during scheduled outpatient clinic visits at least 1 month post-discharge.

The secondary outcomes were operative duration, time to drain and suture removal, and total length of hospitalization.

### 2.4 Statistical analysis

Categorical variables are presented as frequencies and percentages (%) and compared using the chi-squared test or Fisher's exact test, as appropriate. Continuous variables were assessed for normal distribution using the Shapiro–Wilk test. Normally distributed continuous variables were expressed as mean ± standard deviation (SD) and compared using the independent samples *t*-test. Non-normally distributed continuous variables are presented as medians with interquartile ranges (IQR, 25th–75th percentiles) and analyzed using the Mann–Whitney *U*-test.

To mitigate selection bias and balance the baseline characteristics between the SIS and STRS groups, a 1:1 propensity score matching (PSM) analysis was performed. Propensity scores were estimated using a multivariate logistic regression model based on 12 baseline covariates: sex, age, body mass index (BMI), serum albumin level, diabetes, COPD, active smoking status, ASA score, previous abdominal surgery, and wound dimensions (length, width, and depth). Matching was executed using the nearest-neighbor greedy algorithm without replacement, applying a stringent caliper width equal to 0.2 times the standard deviation of the logit of the propensity score.

To identify significant independent predictors of overall and specific postoperative complications, a multivariable binary logistic regression analysis was conducted. All baseline demographic, medical, and surgical variables were initially assessed for inclusion in the multivariable models using univariate logistic regression in a forward selection manner, whereby a *P*-value of less than 0.10 prompted their inclusion. Variables meeting this threshold were subsequently incorporated into the multivariable models, where a *P*-value of less than 0.05 was considered statistically significant. Adjusted odds ratios (aORs) and 95% confidence intervals (CIs) were calculated.

Statistical significance was defined as a two-sided *P*-value of <0.05. All statistical analyses were conducted using R-studio (version 4.0.3) and Jamovi (version 2.3.21) statistical software.

## 3 Results

### 3.1 Characteristics of the study cohort

A total of 89 patients who met the inclusion criteria were enrolled in this study, comprising 39 and 50 patients in the SIS and STRS groups, respectively. There were no statistically significant differences in baseline characteristics between the two groups (Table 1). The median age was 56 years and 65 years in the SIS and STRS group (*P* = 0.329), respectively, median follow up times were 6 months and 5.5 months, respectively (*P* = 0.432); and median BMI were 26 and 24.5, respectively (*P* = 0.059). Regarding comorbidities, the prevalences of diabetes (35.9% vs. 26.0%), chronic obstructive pulmonary disease (7.7% vs. 10.0%), and smoking history (12.8% vs. 16.0%) were not significantly different. Furthermore, the wound dimensions, preoperative albumin levels, and ASA score distribution were comparable between the two groups.

**Table 1:** Patient characteristics.

Characteristic	Unmatched			Matched		
	SIS (*n* = 39^a^)	STRS (*n* = 50^a^)	*P*-value	SIS (*n* = 29^a^)	STRS (*n* = 29^a^)	*P*-value
Age at Surgery	56 (51,69)	65 (52,74)	0.329	56 (51,67)	66 (54,72)	0.331
BMI (kg/m^2^)	25.66 ± 2.57	24.61 ± 2.75	0.068	25.33 ± 2.75	24.84 ± 2.83	0.51
Gender			0.224			0.188
Female	16 (41.0%)	27 (54%)		13 (44.8%)	18 (62.1%)	
Male	23 (59.0%)	23 (46%)		16 (55.2%)	11 (37.9%)	
Diabetes	14 (35.9%)	13 (26.0%)	0.358	8 (27.6%)	8 (27.6%)	1
COPD	3 (7.7%)	5 (10.0%)	1.000	3 (10.3%)	3 (10.3%)	1
Active Smoker	5 (12.8%)	8 (16.0%)	0.768	3 (10.3%)	4 (13.8%)	1
Serum albumin (g/L)	34.57 ± 3.94	33.49 ± 4.24	0.221	34.08 ± 4.07	34.14 ± 4.41	0.956
Wound length (cm)	16.56 ± 5.96	16.40 ± 4.82	0.889	16.62 ± 6.58	16.00 ± 4.30	0.673
Wound width(cm)	4.(4,5)	4 (3,5)	0.445	4 (3,5)	4 (4,5)	0.778
Wound depth(cm)	3 (2,3)	3 (2,3)	0.182	3 (2,3)	3 (2,3)	0.756
Days between SSI and wound salvage	11 (8,13)	10.5 (9,13)	0.796	12 (7,14)	11 (8,13)	0.476
Microbial cultures			0.643			0.246
*S. aureus*	6 (15.4%)	5 (10.0%)		6 (20.7%)	0 (0.0%)	
MRSA^b^	3 (7.7%)	8 (16.0%)		3 (10.3%)	5 (17.2%)	
*Enterococcus spp.*	6 (15.4%)	10 (20.0%)		5 (17.2%)	4 (13.8%)	
*Escherichia coli*	4 (10.3%)	8 (16.0%)		3 (10.3%)	6 (20.7%)	
*Acinetobacter baumannii*	8 (20.5%)	5 (10.0%)		3 (10.3%)	4 (13.8%)	
*Klebsiella pneumoniae*	5 (12.8%)	6 (12.0%)		3 (10.3%)	4 (13.8%)	
others	7 (17.9%)	8 (16.0%)		6 (20.7%)	6 (20.7%)	
ASA Score			0.183			0.597
1	0 (0.0%)	2 (4.0%)		0 (0.0%)	1 (3.4%)	
2	22 (56.4%)	19 (38%)		16 (55.2%)	12 (41.4%)	
3	13 (33.3%)	23 (46%)		9 (31.0%)	11 (37.9%)	
4	4 (10.3%)	6 (12.0%)		4 (13.8%)	5 (17.2%)	
Previous Abdominal surgery			0.944			0.346
Acute abdominal trauma	16 (41.0%)	18 (36.0%)		6 (20.7%)	3 (10.3%)	
Elective digestive tract surgery	7 (17.9%)	12 (24.0%)		11 (37.9%)	8 (27.6%)	
Elective hepatobiliary surgery	7 (17.9%)	10 (20.0%)		3 (10.3%)	9 (31.0%)	
Elective gynecological surgery	3 (7.7%)	4 (8.0%)		6 (20.7%)	6 (20.7%)	
Others	6(15.4%)	6(12.0%)		3 (10.3%)	3 (10.3%)	

a Median (Q1, Q3); *n* (%).

b MRSA, Methicillin-resistant Staphylococcus aureus.

After a 1:1 propensity score matching, 29 matched pairs (58 patients) were identified. The matching process successfully eliminates baseline discrepancies. In the matched cohort, all patient demographics, comorbidities, and wound dimensions—including wound length, width, and depth—were highly comparable with no statistically significant differences (all *P* > 0.05), ensuring a balanced comparison for clinical outcomes.

Microbiological analysis of the infected wounds prior to secondary closure revealed that the most frequently isolated pathogens across the entire cohort were *Enterococcus spp.* (18%), *Acinetobacter baumannii* (14.6%), and *Escherichia coli* (13.5%). There was no significant difference in the microbiological profile or proportion of multidrug-resistant organisms between the SIS and STRS groups (*P* > 0.05).

### 3.2 Postoperative complications

Regarding primary outcomes, the overall complication rate (any complication) was significantly lower in the STRS group compared to that in the SIS group (16% vs. 46.2%, *P* = 0.002). Nine patients (23.1%) in the SIS group developed recurrent SSI, whereas three patients (6.0%) in the STRS group showed a statistically significant difference (*P* = 0.028). Correspondingly, the reoperation rate was significantly higher in the SIS group (17.9%) than in the STRS group (4.0%) (*P* = 0.039). Post-matching analysis revealed that STRS was associated with a lower overall complication rate. Although specific localized complications, such as recurrent SSI (6.9% vs. 17.2%, *P* = 0.423) and the need for reoperation (3.4% vs. 17.2%, *P* = 0.194), showed a notable trend toward reduction in the STRS group, they did not reach statistical significance in the matched cohort, likely because of the reduced sample size post-matching.

Although the incidences of wound dehiscence, seroma, and fat liquefaction were higher in the SIS group, the differences were not statistically significant (*P* > 0.05) in either the pre- or post-matching analyses. The incidence of hematoma was similar between the two groups (Table 2).

**Table 2:** Primary outcomes.

Characteristic	Unmatched			Matched		
	SIS (*n* = 39^a^)	STRS (*n* = 50^a^)	*P*-value	SIS (*n* = 29^a^)	STRS (*n* = 29^a^)	*P*-value
Any complication	18 (46.2%)	8 (16.0%)	0.002^b^	12 (41.4%)	5 (17.2%)	0.043^b^
Recurrent SSI	9 (23.1%)	3 (6.0%)	0.028^b^	5 (17.2%)	2 (6.9%)	0.423
Wound breakdown	5 (12.8%)	2 (4.0%)	0.233	4 (13.8%)	1 (3.4%)	0.352
Hematoma	2 (5.1%)	2 (4.0%)	1.000	2 (6.9%)	2 (6.9%)	1
Seroma	5 (12.8%)	1 (2.0%)	0.085	2 (6.9%)	1 (3.4%)	1
Fat necrosis	6 (15.4%)	2 (4.0%)	0.131	3 (10.3%)	1 (3.4%)	0.611
Reoperation	7 (17.9%)	2 (4.0%)	0.039^b^	5 (17.2%)	1 (3.4%)	0.194

a *n* (%).

b Statistically significant.

### 3.3 Secondary outcomes

In both unmatched and matched comparisons, the operative duration of the secondary suture in the STRS group was significantly longer than that in the SIS group (24 [20,27] min vs. 20 [15,21] min, 24 [22,25] min vs. 20 [15,20] min, respectively; *P* < 0.05). The total length of hospitalization for patients in the STRS group was significantly shorter than that in the SIS group, with medians of 17.5 days vs. 21 days and 18 days vs. 22 days, respectively (*P* < 0.05). There were no statistically significant differences between the two groups regarding time to drain (3 [2,3] days vs. 2 [2,3] days, 3 [2,3] days vs. 3 [2,3] days, respectively *P* > 0.05) and suture removal (12 [11,13] days vs. 12 [11, 12] days, 12 [11,12] days vs. 12 [11, 12] days, respectively *P* > 0.05) (Table 3).

**Table 3:** Secondary outcomes.

Characteristic	Unmatched			Matched		
	SIS (*n* = 39^a^)	STRS (*n* = 50^a^)	*P*-value	SIS (*n* = 29^a^)	STRS (*n* = 29^a^)	*P*-value
Surgery duration	20 (15,21)	24 (20,27)	<0.001^b^	20 (15,20)	24 (22,25)	<0.001^b^
Time to drain removal	2 (2,3)	3 (2,3)	0.355	3 (2,3)	3 (2,3)	0.368
Time to suture removal	12 (11,12)	12 (11,13)	0.549	12 (11,12)	12 (11,12)	0.816
Length of hospitalization	21 (18,26)	17.5 (15,20)	<0.001^b^	22 (19,26)	18 (15,20)	<0.001^b^

a Median (Q1, Q3).

b Statistically significant.

### 3.4 Predictors of postoperative complications

Univariate logistic regression was first performed to screen potential predictors for postoperative outcomes. Variables meeting the *P* < 0.10 threshold were subsequently evaluated in multivariable binary logistic regression models. (See Table, Supplemental Digital Content, which shows the multivariable analysis for all complications.)

In the multivariable analysis for overall complications (AUROC = 0.68, overall *P* = 0.002), the STRS method was identified as a significant independent protective factor. Patients who received STRS had a significantly lower risk of overall complications compared to those who received standard interrupted suture (SIS) (aOR, 0.22; 95% CI, 0.08–0.59; *P* = 0.003).

Subgroup analyses of specific complications yielded similar protective trends for the STRS technique. STRS was the sole independent protective predictor against recurrent surgical site infection (SSI) (aOR, 0.21; 95% CI, 0.05–0.85; *P* = 0.028). Furthermore, STRS significantly reduced the need for reoperation (aOR, 0.17; 95% CI, 0.03–0.94; *P* = 0.042). Age demonstrated a marginal trend in the reoperation model but did not achieve strict statistical significance (aOR, 0.95; 95% CI, 0.91–1.00; *P* = 0.051).

Regarding seroma formation (model AUROC = 0.83, overall *P* = 0.007), active smoking was identified as a strong independent risk factor, increasing the odds of seroma by over 10-fold (aOR, 10.50; 95% CI, 1.54–71.44; *P* = 0.016). The STRS method showed a marginal trend toward protecting against seroma (aOR, 0.10; 95% CI, 0.01–1.02; *P* = 0.052). Notably, the variable “previous abdominal surgery” was excluded from this specific multivariable model due to complete statistical separation (zero seroma events in specific subgroups).

For fat necrosis (model AUROC = 0.77, overall *P* = 0.038), while the overall model was significant, neither STRS (aOR, 0.21; *P* = 0.077) nor age (aOR, 0.96; *P* = 0.085) reached the strict threshold of statistical significance (*P* < 0.05) as independent predictors. Finally, due to the limited number of events, no candidate variables met the univariate inclusion criteria (*P* < 0.10) for wound breakdown/dehiscence and hematoma, precluding further multivariable modeling for these endpoints.

## 4 Discussion

SSI remain a frequent and burdensome complication of abdominal surgery (1, 12), significantly contributing to postoperative morbidity, mortality, and healthcare costs (13). Despite the existence of various guidelines, recent assessments indicate that abdominal wound closure techniques and SSI prevention strategies vary widely among surgeons and lack a standardized approach for high-risk cases (3). For secondary closure of infected incisions, these challenges are exacerbated by the induction of severe tissue edema and fragility. Furthermore, the necessity for prolonged open drainage inevitably leads to skin edge retraction and fibrosis. Consequently, the incision experiences extreme tension during secondary closure. Excessive tension directly predisposes the wound to mechanical dehiscence and critically compromises local microcirculation. The resulting ischemia and hypoxia at the skin edges inhibit fibroblast proliferation and collagen synthesis, while impairing local immune defense, thereby creating a vicious cycle that increases the risk of SSI recurrence and wound breakdown.

Surgeons aim to achieve optimal wound healing. Although traditional interrupted suturing is simple, it can produce a “cutting” effect on the skin edges, and tension is concentrated at the incision margin, easily causing circulatory compromise (14). Contrastingly, the modified buried vertical mattress suture technique used in this study has significant biomechanical advantages. Through deep needle entry and a specific suture trajectory, STRS transfers the tension load of the incision to the deep subcutaneous connective tissue, allowing the skin edges to oppose in a tension-free or low-tension state (7). This redistribution of tension may help mitigate the risk of ischemia and necrosis of skin edges. STRS appears to be advantageous for facilitating closure of the subcutaneous dead space. Fluid accumulation in the dead space (seroma) serves as a breeding ground for bacteria. Although the crude incidence of seroma trended lower in the STRS group compared to the SIS group (2.0% vs. 12.8%), our multivariable analysis revealed that active smoking was the primary independent risk factor for seroma formation (aOR = 10.50), while the STRS technique demonstrated a marginal protective effect (*P* = 0.052). This reduction in dead space and fluid accumulation likely contributes to the lower SSI recurrence rate in the STRS group. STRS facilitates moderate eversion of skin edges, increasing the contact area of the dermal layer, which is conducive to microvascular regeneration and connection (15). Importantly, subcutaneous tension-reduction suturing helps avoid lateral compression caused by traditional interrupted sutures, thereby potentially preserving compromised marginal skin perfusion (14).

When managing infected abdominal incisions, the choice of reconstructive modality is guided by the classic reconstructive ladder (16) and overarching goals of functional abdominal wall reconstruction (17). Although split-thickness skin grafting is a viable fallback option for massive defects for which direct closure is physically impossible or has failed, it is generally considered suboptimal for definitive abdominal wall reconstruction. Skin grafts lack the durability, elasticity, and subcutaneous padding of native tissue, often leading to contour deformities, vulnerability to trauma, and dense adhesion to underlying structures. Therefore, restoring the dynamic integrity and native protective barrier of the abdominal wall through direct fascial and epidermal approximation is universally prioritized when it is biomechanically feasible. In this context, STRS serves as a crucial adjunct to safely expand the indications for direct closure, mitigating the extreme tension that would otherwise necessitate a skin graft or complex flap reconstruction. It is important to emphasize that specific complex indications such as extensive fascial defects, exposed bowel, exposed surgical meshes, or massive skin deficits dictate the fundamentally different reconstruction algorithms. Based on our study design, extreme scenarios were excluded from this cohort to ensure homogeneity. For the deep and superficial incisional SSIs targeted in our study, utilizing STRS to achieve definitive native tissue closure provided the optimal balance of functional restoration and infection control.

The remarkable reduction in SSI recurrence (from 23.1% to 6.0%) observed in the STRS cohort-further confirmed by our multivariable logistic regression as an independent protective factor (aOR = 0.21)- underscores the critical intersection between tissue biomechanics and wound immunity. Traditional interrupted sutures often concentrate mechanical stress directly at the incision margins, causing a localized “strangulation” effect that compromises capillary perfusion. In contrast, the modified buried vertical mattress technique (STRS) employed in this study anchors into the deep subcutaneous connective tissue, effectively uncoupling the epidermal edges from the underlying mechanical pull. Recent biomechanical studies corroborate that such active tension redistribution prevents microvascular collapse and continuous profibrotic factor (e.g., TGF-β) secretion at the wound edge (18). By securing a well-perfused and low-tension microenvironment, local immune defenses are preserved, which provides a potential mechanistic explanation for the reduced infection rates observed in our STRS patients.

Although the operative time in the STRS group was significantly longer than that in the SIS group (median extension of 4 min), this was primarily attributed to the complexity of the technique, requiring more precise suturing. However, considering the significant reduction in total hospital stay (shortened by approximately 3.5 days) and the multivariable-adjusted independent reduction in reoperation risk (aOR = 0.17), this minor time investment is highly clinically cost-effective. For patients, avoiding reoperation and shortening hospitalization mean less suffering and a lower economic burden. Although STRS showed a lower incidence of minor complications such as wound breakdown, fat necrosis, and hematoma, these differences did not achieve strict statistical significance as independent predictors. As evidenced by our multivariable regression attempts, the notably low event rates for severe mechanical failure (dehiscence) and bleeding (hematoma) precluded robust predictive modeling. This limitation likely reflects the overall effectiveness of contemporary wound care, suggesting that future large-scale, multicenter cohorts are required to statistically unmask the subtle protective nuances of STRS on these specific minor complications. However, the consistent trend favoring STRS is clinically relevant and aligns with previous reports that demonstrated improved outcomes with tension-modifying suturing techniques in high-risk or infected wounds.

Notably, all participants underwent NPWT before wound repairment. Although NPWT effectively promoted granulation tissue growth and drainage, conclusions in the literature regarding its ability to reduce SSI recurrence after secondary closure vary (19, 20). Hence, closed-incision NPWT (ciNPWT) was not applied post-closure, as reinfection is believed to originate primarily from residual bacteria within the wound rather than external contamination. Further randomized controlled trials are required to clarify the role of ciNPWTs in high-risk abdominal wounds.

This study has several limitations. **First**, its retrospective, non-randomized design inherently introduces a selection bias. Although we employed propensity score matching (PSM) to rigorously balance baseline covariates, inherent chronological bias remained. Because STRS was increasingly adopted over the four-year study period and gradually replaced conventional closure, the SIS group essentially served as a historical control. Consequently, longitudinal advancements in perioperative care and targeted antibiotic stewardship may have independently contributed to the improved outcomes. To overcome this, future prospective multi-center randomized controlled trials (RCTs) are imperative to robustly isolate the therapeutic efficacy of STRS. Furthermore, there is a potential for indication bias. As STRS was preferentially applied to high-tension wounds with severe skin retraction, these unmeasured severity variables could not be fully adjusted for in our statistical models. However, it is noteworthy that if STRS was used in more severe cases, the observed benefits in the STRS group might actually represent an underestimation of its true efficacy. Additionally, outcome assessments were performed by the treating surgical team without blinding, introducing a risk of detection bias. Although we utilized standardized CDC criteria to minimize subjectivity, the lack of an independent adjudication committee remains a methodological constraint. **Second**, our cohort was restricted to patients with superficial SSIs. Given the anatomical complexity and severity of abdominal wound infections, it remains unclear whether STRS is safe and applicable to deep-incisional SSIs involving extensive fascial defects or complex organ/space SSIs. Further investigation is required to define the anatomical boundaries for their application. **Finally**, our outcome assessment was limited to short-term postoperative complications. Long-term functional and aesthetic outcomes, such as scar morphology, abdominal wall contour, and overall patient satisfaction, were not evaluated. Incorporating patient-reported outcome measures (PROMs) in future longitudinal studies is essential for comprehensively assessing the holistic benefits of this reconstructive approach.

## 5 Conclusion

During the repair of infected abdominal incisions, using STRS techniques for secondary closure is associated with lower rates of recurrent infection and reoperation, and a significantly shorter hospital stay compared to conventional suturing methods. Despite a slightly prolonged operative time, the clinical benefits were significant. Future large-sample, multicenter, randomized controlled trials are recommended to further validate the application value of this technique in the management of complex abdominal wounds.

## Data Availability

The raw data supporting the conclusions of this article will be made available by the authors, without undue reservation.
